# The Radial Bow following Square Nailing in Radius and Ulna Shaft Fractures in Adults and its Relation to Disability and Function

**DOI:** 10.5704/MOJ.1607.003

**Published:** 2016-07

**Authors:** MB Dave, KD Parmar, BA Sachde

**Affiliations:** PDU Government Medical College, Rajkot, Gujarat, India

**Keywords:** Radial bow, square nails, radius and ulna shaft fractures, supination pronation arc, DASH score

## Abstract

One of the points made against nailing in radius and ulna shaft fractures has been the loss of radial bow and its impact on function. The aims of the study were to assess the change in magnitude and location of the radial bow in radius and ulna shaft fractures treated with intramedullary square nails and to assess the impact of this change on functional outcome, patient reported disability and the range of motion of the forearm. We measured the magnitude of radial bow and its location in the operated extremity and compared it to the uninjured side in 32 adult patients treated with intramedullary square nailing for radius and ulna shaft fractures at our institute. The mean loss of magnitude of maximum radial bow was 2.18 mm which was statistically significant by both student-T test and Mann-Whitney U test with p value less than 0.01. The location of maximum radial bow shifted distally but was statistically insignificant. The magnitude of maximum radial bow had a negative correlation with DASH score that was statistically insignificant (R=- 0.22, p=0.21). It had a positive, statistically significant correlation to the extent of supination in the operated extremity (R = 0.66, p = 0.0004). A loss of up to 2mm of radial bow did not influence the functional outcome as assessed by criteria reported by Anderson et al. The magnitude of radial bow influenced the supination of the forearm but not the final disability as measured by DASH score. Intramedullary nailing did decrease the magnitude of radial bow but a reduction of up to 2mm did not influence the functional outcome.

## Introduction

Radius and ulna shaft fractures are common in the adult population. Historically, the recommendation is for open reduction and internal fixation of forearm bone fractures for acceptable functional results 1,2. In our institute, intramedullary nailing with square nails is quite a frequent operative option for radius and ulna shaft fractures in adult population, with acceptable results. Open reduction and plating of forearm bone fractures is fraught with complications like skin and wound healing problems, implant related problems, infections, peri-implant fractures, soft tissue and tendon adhesions and refractures following implant removal^3-5^. One of the arguments in favour of open reduction and plating has been the avoidance of rotational malalignment and maintenance of radial bow^6^. With this study, we aimed to ascertain the changes to radial bow with intramedullary nailing of shaft fractures regarding its location and magnitude and its impact on supinationpronation range of movement and the functional outcome.

## Materials and Methods

In this study, 35 adult patients with radius and ulna shaft fractures who consented for participation and met the exclusion/inclusion criteria were treated with intramedullary square nailing at our institute.

Inclusion criteria were: age more than 18 years, closed radius and ulna shaft fractures with adequate medullary cavity, no previous initial treatment. Exclusion criteria were other fractures in the same or opposite extremity, open fractures and segmental fractures.

Under regional or general anesthesia, supine position and use of image intensification, closed reduction and fixation by intramedullary square nails (Nebula Surgical, Guj, India) were performed. Open reduction through a small incision was resorted in six cases when closed nailing was not possible. Appropriate size nails with largest possible diameter were selected. An above elbow dorsal slab was applied for two weeks followed by a full above elbow cast for four weeks or shorter period when signs of sticky union were visible on radiographs. After removal of the cast, all patients were given gradual physiotherapy. All patients were followed up with serial radiographs till radiological union was achieved, after which the location of the radial bow was identified and its magnitude *(a,a’)* measured and compared with opposite side.

**Fig. 1:**
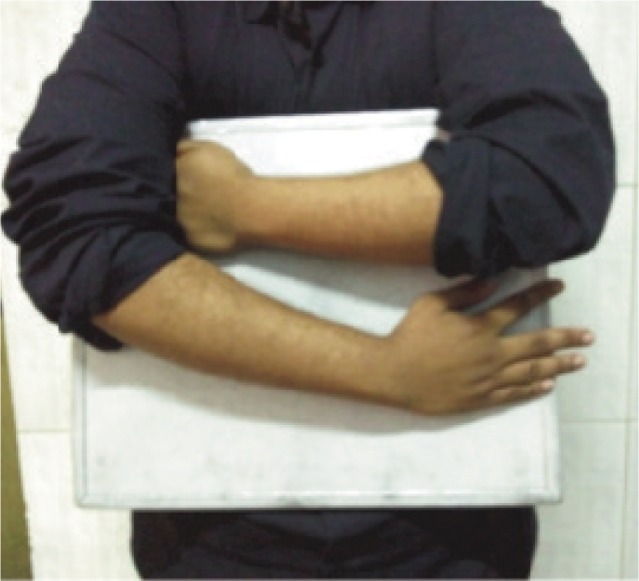
The technique for simultaneous forearm radiography with internal rotation at shoulder.

We used a technique for taking the anteroposterior radiograph with internal rotation at both shoulders and simultaneous radiographs of both forearms to avoid overlapping images of radius ulna and identical measurements for radial bow (Figure 1). This technique proved extremely helpful in forearms with limited supination.

**Fig. 2:**
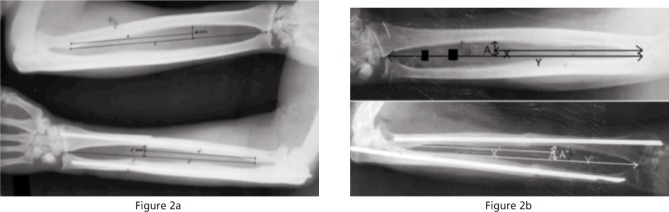
Calculation of radial bow location and magnitude compared to normal limb.

The location of the radial bow was calculated by a ratio of length of segment from midpoint of the bicipital tuberosity to location of maximum radial bow *(x,x’)* and the length of radius from midpoint of bicipital tuberosity to the most ulnar point of distal radius *(y,y’)* on the radiographs of both forearms taken in identical position (Figures 2a, 2b).

DASH scores and range of pronation supination movements were measured at radiological union and at six weeks following clinical union. Final functional outcomes were measured based on criteria of Anderson *et al*^7,8^ (Table I).

**Table I:** Criteria for functional outcome as proposed by Anderson *et al.*

Result	Union	Flexion and extension at wrist joint (the loss of function in degree)	Supination and Pronation (the loss of function in %)
Excellent	Present	<10° loss	<25% loss
Satisfactory	Present	<20° loss	<50% loss
Unsatisfactory	Present	<30° loss	>50% loss
Failure		Non-union with or without loss of motion	

Statistical analysis was done with both parametric and non-parametric tests for comparing magnitude and location of radial bow in normal and operated forearm. Pearson correlation coefficient and Spearman’s Rho test were used to assess relationship between magnitude of radial bow in injured extremity and DASH scores and ranges of supination and pronation. Finally, Chi Square Test was used to assess correlation between loss of radial bow and functional outcomes.

## Result

Thirty-two patients (21 male and 11 female) with mean age of 34.6 ±12.8 years were available for complete follow up till six weeks following radiological union. Two patients were lost to follow up and one patient required conversion to plating and bone grafting for persistent non-union and was excluded from the analysis. Two patients (6.25%) required bone grafting for union and one patient (3.12%) had infection that subsided with antibiotics. Mean time to union was 11.25 weeks (SD ±1.6 weeks).

At six weeks following radiological union, the mean DASH score was 17.6 (with SD ±10.4). Applying the criteria of Anderson *et al*, 15 patients (46.87%) had excellent outcome, 15 patients (46.87%) had satisfactory outcome, and two patients (6.25%) had unsatisfactory outcome.

Mean maximum radial bow *(a)* in (opposite) normal forearm was 11.3mm (SD ±1.9mm) and that in operated extremity *(a’)* at fracture union was 9.1mm (SD ±1.7mm). The mean loss of the extent of maximum radial bow was 2.18mm which was statistically significant by both student T test and Mann-Whitney U test, with p value less than 0.01. There was loss of maximum radial bow with radius and ulna nailing in shaft fractures.

The ratio for location of maximum radial bow in normal forearm had a mean value of 62.3% while that in operated forearm was 65.24%, indicating a distal migration of location of maximum radial bow in operated extremity but this was statistically non-significant (p >0.05). The location of maximum radial bow shifted distally with radius and ulna nailing but was not statistically significant in our study.

The magnitude of radial bow *(a’)* was correlated to DASH score and the amount of supination and pronation in the operated arm using Pearson correlation coefficient and Spearman’s Rho test. The amount of radial bow had a negative correlation with DASH score that was not statistically significant (R= - 0.22, p=0.21). The magnitude of radial bow had a positive, statistically significant correlation to amount of supination in the operated extremity (R = 0.66, p = 0.0004), however it had weaker and statistically insignificant correlation with amount of pronation (R =0.138, p=0.451). Thus the magnitude of radial bow has positive influence on the amount of supination achieved in fractured limb.

Finally, on use of Chi Square Test to correlate a loss of radial bow and functional outcome, the p-value was 0.12, statistically not significant at p < 0.05. A loss of up to 2mm of radial bow did not influence the functional outcome in our study.

## Discussion

Traditionally, open reduction and internal fixation of radius and ulna fractures have been considered the gold standard. However, open reduction and internal fixation for both bone fractures entails complications of soft tissue healing and compartment syndrome^4^. Moreover, plate fixation has inherent issues of stress shielding, peri-implant fractures, soft tissue and tendon adhesions or attrition. Refracture rates after implant removal are also high^3,5^ and it entails additional surgery and further period of immobilization or caution.

We at our institute have been frequently using intramedullary square nails for adult patients with radius and ulna fractures. There have been many reports of good results of such fixation in paediatric patients; however, studies in adult population are limited. Intramedullary fixation has many advantages: less blood loss, smaller scars, faster union and fewer infections. However, it has drawbacks of not allowing immediate mobilization and concerns about rotational stability and maintenance of reduction. Moreover, they cannot be used in patients with very narrow medullary cavity. Square nails of appropriate thickness in a round medullary cavity do give some rotational stability.

Another point made against nailing has been the loss of radial bow and its impact on function. Radius and ulna have a complex anatomic relationship and their relative lengths and curves are necessary for full range of supination and pronation movements. The elbow and wrist joints also do not allow much compensation for the loss of these movements but shoulder joint enjoys considerable freedom and may compensate for the loss functionally. Schemitsch *et al* studied, in cadaveric models, the effect of plating and nailing on radial bow and angulation^9^. They concluded that the magnitude of maximum radial bow and the radial angulation were changed by nailing both forearm bones after osteotomy (p < 0.05). Despite this, in their study, they stated that both plating and nailing achieved reductions which were well within the limits of what is radiographically acceptable. This loss of radial bow and its impact on outcome was studied by Schemitsch and Richards by studying malunion of radial fractures concluded that the magnitude of radial bow and its location did affect forearm rotations and values of radial bow near normal and also location of radial bow near normal led to forearm rotations comparable to normal side^10^. In our study also, the magnitude of radial bow had positive, statistically significant relation to the supination achieved. The nearer to normal restoration of radial bow, the more were the chances of achieving full supination. It had a lesser impact on pronation which was statistically not significant and a negative statistically insignificant relation to DASH scores.

Street *et al* reported a 93% union rate with the use of square nails in forearm fixation^11^. Ozkaya *et al* compared interlocked intramedullary nailing with plating and reported shorter operative times and shorter time to union in the nailing group. The two groups did not differ in terms of functional outcome and DASH scores^12^. The union rates (32/35) and time to union in our study are comparable to these and other studies. Functional results were also comparable to studies by Moerman *et al*^13^, Chapman *et al*^14^and Anderson *et al*^7,8^.

Plate osteosynthesis versus intramedullary nailing for fractures of both forearm forearm bones fractures were compared by Lee SK *et al*^15^ and they concluded that plate osteosynthesis resulted in a an excellent extent of restoration to the conditions prior to the injury. They also observed that such significant differences in the restoration of the radial bow had no effect on the final clinical outcome. In properly selected patients, their results suggested that intramedullary nailing can be acceptable and effective treatment option for fractures in both forearm bones. Our study shows that a loss of up to 2mm of magnitude of radial bow did not affect the functional outcome.

## Conclusion

Intramedullary square nailing of radius and ulna shaft fractures in adult patients gives satisfactory functional outcomes in properly selected cases. The magnitude of radial bow influences the supination of the forearm but not the patient reported disability. Intramedullary nailing does decrease the magnitude of radial bow but a reduction of up to 2mm of radial bow does not influence the functional outcome.
